# Burden and Correlates of HIV among Men Who Have Sex with Men in West Bengal, India: Analysis of Sentinel Surveillance Data

**DOI:** 10.1371/journal.pone.0127232

**Published:** 2015-05-21

**Authors:** Tanmay Mahapatra, Subrata Biswas, Srijita Nandi, Piyali Ghosh, Mallika Ghosh, Soumya Mondal, Malay K. Saha

**Affiliations:** 1 National Institute of Cholera and Enteric Diseases, P-33, Scheme—XM, Beliaghata, Kolkata, West Bengal, India; 2 Monitoring and Evaluation Division, West Bengal AIDS Prevention and Control Society, Salt Lake, Kolkata, West Bengal, India; The University of New South Wales, AUSTRALIA

## Abstract

**Background:**

Little is known about the socio-behavioral risk factors for HIV acquisition among hard-to-reach men who have sex with men (MSM) population in India, particularly from the densely populated eastern part. Thus to measure the burden and correlates of HIV among MSM in West Bengal state of eastern India, a cross-sectional analysis of the national HIV Sentinel Surveillance (HSS) data was conducted.

**Methods:**

In 2011, between July and September, involving all sentinel sites of the state, 1237 consenting MSM were anonymously interviewed and tested for HIV following national guidelines. Using a short, structured questionnaire, information was collected on socio-behavioral factors along with sexual practices and was analyzed to determine burden of HIV and the role of its socio-behavioral correlates on HIV acquisition.

**Results:**

Among participants, mean age was 23.4 years, 44.55% were “Kothis” (usually receptive partner) and 25.1% admitted receiving money for sex with man. HIV sero-positivity was 5.09%. Using logistic regression method, for both bivariate and multivariate (with saturated model) analyses, transport-workers [adjusted odds ratio (AOR)=8.95, 95% confidence interval (95%CI): 1.09-73.71), large business-owners/self-employed (AOR=8.46, 95%CI: 1.25-57.49), small business-owners/cultivators (AOR=7.90, 95%CI: 1.67-37.38), those who visited the sentinel site for official purposes (AOR=7.60, 95%CI: 1.21-47.83) and paying money for having sex with men (AOR=3.03, 95%CI: 1.10-8.33) were strongly associated with higher HIV sero-positivity with than their counterparts. Using the parsimonious model for multivariate analysis, Kothis (AOR=4.64, 95%CI: 1.03-20.89), paying (AOR=2.96, 95%CI: 1.15-7.58) or receiving (AOR=2.06, 95%CI: 1.06-3.99) money for having sex with a man were associated with higher risk of HIV.

**Conclusions:**

Focused intervention targeting the high risk MSM subgroups including Kothis, transport-workers, business-owners/self-employed and those who exchanged money for having sex with men, seemed to be the need of the hour for preventing the spread of HIV infection within and from this understudied population.

## Introduction

India has been facing a major public health challenge with an estimated 2.09 million HIV infected population, the 3^rd^ highest globally [1–3]. In the era of changing dynamics of HIV epidemic, although predominant mode of transmission in the country still remains heterosexual, the epidemic is now being concentrated among high risk groups like MSM [2]. Overall, 1.5% of all HIV positive cases reported homosexual activity in India during 2011 [2,4]. Indian MSM are diverse group of people without a distinct homosexual identity like western countries [5–7]. They are highly stigmatized, often targeted for social discrimination or abuse [8,9] and hence are mostly hidden and marginalized [5–7,10,11]. Due to their diverse sexual mixing pattern, poor access to awareness programs and less utilization of control measures, MSM in India are at higher risk of sexually transmitted infections including HIV [2,7,12]. Estimated HIV prevalence among MSM in India was 7.3% in 2009 against the adult prevalence of 0.31% [2,4]. In West Bengal (fourth populous state of India and the seventh-most populous sub-national entity in the world) [13], the estimated HIV prevalence among MSM was 5.1% during 2011 against the adult HIV prevalence being 0.29% [1,2,12]. This disproportionately higher (17.6 times) prevalence is a key concern and the scenario might be more problematic as the overall coverage of targeted intervention (TI) program to provide treatment, care and support to this hard-to-reach high risk group was estimated to be about 64% during 2011 [2]. The most common self-perceived categories of MSM identified in India are Kothi (commonly receptive), Panthi (usually insertive) and Double-decker (get engaged in both roles) [6,7,10,14]. Under socio-cultural pressure, many MSM get married to females and engage in heterosexual activity keeping their homosexual behavior secret. As a result, the risk of HIV acquisition among the unassuming partners of married MSM increases, leading to a rising threat of HIV catastrophe for otherwise low-risk general population [7]. In a country like India, where husbands’ sexual behavior is one of the most important contributing factors for HIV acquisition risk of the married women [15], the potential of MSM to become a hidden bridge population between high risk groups and general people needs to be understood also.

Analytical research to understand the socio-demographic and behavioral correlates of HIV acquisition is thus urgently required for designing culturally appropriate multi-level risk reducing intervention strategies to reduce HIV transmission within and from MSM population in India. This is particularly pertinent in states like West Bengal, where HIV prevalence among this population is remaining persistently high [12]. To our knowledge, to date, hardly any such study has explored the socio-behavioral correlates of HIV risk among MSM in this state. This article endeavors to build the scientific evidence by reporting socio-demographic and behavioral correlated of HIV risk among MSM by analyzing HSS data for the state of West Bengal.

## Methods

### Ethics statement

HIV sentinel surveillance (HSS) was conducted following Unlinked Anonymous Testing strategy approved by Ethical Committee of National AIDS Control Organization, New Delhi. The study involving human participants is in compliance with the Helsinki declaration. Prior to the interview and sample collection, details of the study were explained to the subjects in a language that they understood completely and voluntary written informed consents were obtained from each and every subject maintaining confidentiality as per the standard national guidelines. The written informed consent procedure for minor includes both assent by the participant and consent from the next to kin, caretakers, or guardian on behalf of the minors. Subjects were free to decline participation without any consequences towards their treatments. Data were securely preserved with confidentiality. The study was approved by the Institutional Ethics Committee of National Institute of Cholera and Enteric Diseases (No. A-1/2013-IEC).

### Study design

A cross-sectional analysis of HSS 2010–11 data from all five sentinel sites, one each in Kolkata, Hooghly, Burdwan, Nadia and Darjeeling districts for MSM in West Bengal was conducted. Men aged between 15–49 years who had anal or oral sex with a male partner in the last one month and visited designated HSS sites (drop-in-center where MSM meet, interact with them, strive for advice, seek healthcare services and collect condoms) in West Bengal during 2010–11 round of HSS were eligible. After obtaining informed consent, recruitment was done according to the order in which eligible MSM attended the sentinel site for the first time (consecutive sampling) [16] during July to September, 2011. HSS was conducted as per the unlinked anonymous testing strategy approved by the Ethical Committee of National AIDS Control Organization, New Delhi.

### Data collection

As per the National AIDS Control Organization (NACO) guidelines, the recruitment continued until 250 eligible MSM were sampled consecutively or 3 months period was over [16]. Combining all 5 sites, a total of 1237 eligible MSM were recruited. Information on socio-demographic (age, current place of residence, years of stay in current place of residence, literacy status, occupation) and behavioral characteristics (reason for coming to the site, self-identified types of MSM, history of injectable drug use (IDU), having sex with female in last 6 months, days since last sex with a man and received/paid money for sex with man in last 1 month) were collected through interviewer administered anonymous interviews using structured and pre-tested questionnaire.

### HIV testing

Unlinked anonymous HIV testing was conducted using dried blood spot sample [16]. Each sample was labeled with sentinel site code, sub-site number, sample number and date of collection without mentioning personal identifiers for maintaining the anonymity of participants. Samples were screened for HIV using sensitive ELISA (Microlisa HIV; J Mitra and Company Pvt Ltd, New Delhi, India). Samples positive for screening were retested using another ELISA (Genedia HIV ½ ELISA 3.1, Green Cross Medical Science Corporation, Chungbuk, South Korea). Samples positive for both the ELISA were considered as HIV sero-positive as per the NACO guidelines of two test strategy for sentinel surveillance [16]. For quality assurance, all the HIV positive and 5% of HIV negative samples were retested at designated National HIV Reference Laboratory [16].

### Data analysis

A descriptive analysis was conducted to understand the distribution of socio-behavioral characteristics and to estimate the burden of HIV among MSM. Bi-variate logistic regression analysis was performed to estimate individual associations between socio-behavioral characteristics and HIV sero-status. Finally, multivariate logistic regression analyses were conducted using two types of model. First, by using the saturated model, association between each socio-behavioral factor and HIV sero-positivity was determined adjusting for all others. Then in the final parsimonious model, on the basis of both prior knowledge from available evidences in contemporary literature regarding the role of potential confounders and the findings from the bivariate analyses, using backward model selection method with constraints, associations between sexual risk behaviors (self-identified type of MSM, having sex with female in last 6 months, having received/paid money for having sex with man in last 1 month and days since last sex with a man) and HIV sero-positivity were determined after controlling for potential confounders (age, current place of residence, literacy status, occupation, years of stay in current place of residence and reasons for coming to the site) identified from background literature. The statistical analyses were done using SAS version 9.3.

## Results

Among all the 1237 participating MSM, the mean age was 23.4 years, 76.6% lived in urban areas, 4.2% were illiterates, 23.4% were students, 33.9% attended the site to avail treatment/counseling, 44.55% were Kothi, 46.83% Double-decker, only 8.6% Panthi, 68.8% had sex with a female within last 6 months, 25.1% admitted receiving money while having sex with a man during last month, none reported using IV drugs and 5.09% were found to be HIV sero-positive (Table 1).

**Table 1:** Distribution of socio-demographic, behavioral factors and HIV sero-status among MSM attending HIV sentinel sites in West Bengal, 2010–11 (N = 1237).

Continuous variable		Mean	Variance
Age (in yrs)		23.4	41.02
Duration of stay in current place of residence (in yrs)		22.2	52.65
Days since last sex with a man (missing = 1)		8.44	97.84
Categorical variable^a^	Categories	N	%
Age group	Very young (15-20yrs)	520	42.04
	Young (21–30 yrs)	570	46.08
	Older (31-49yrs)	147	11.88
Current place of residence	Urban	946	76.60
	Rural	289	23.40
Literacy status	Illiterate	52	4.21
	Up to 5th standard	220	17.80
	6th to 10th standard	577	46.68
	11th standard to Graduation	362	29.29
	Post-graduation	25	2.02
Occupation	Unemployed	163	13.19
	Laborer/Servants	273	22.09
	Transport worker	29	2.35
	Skilled/Semi-skilled worker	123	9.95
	Student	289	23.38
	Small business/cultivation	182	14.72
	Govt./Private Service	144	11.65
	Large business/Self-employed	33	2.67
Reason for coming to the site	Collect condoms	144	11.72
	For official work	10	0.81
	Drop in center/Meet other MSM	344	27.99
	Recreational purpose	314	25.55
	Treatment/Counseling	417	33.93
Self-identified types	Panthi^b^	106	8.62
	Double decker^c^	576	46.83
	Kothi^d^	548	44.55
Had sex with female in last 6 mths	No	385	31.17
	Yes	850	68.83
Received or paid money for having sex with a man in last 1 mth	No	750	60.63
	Paid money	64	5.17
	Received money	311	25.14
	Both	112	9.05
IV drug use in last 1 yr	No	1224	100.00
	Yes	0	0.00
HIV test result	Negative	1174	94.91
	Positive	63	5.09

^a^ Category excludes missing values

^b^ Panthi: Self-identified subgroup of MSM who are externally more masculine, and usually play the insertive partner’s role during anal intercourse with another MSM^14^

^c^Double decker: Self-identified subgroup of MSM whose gender identity is often neutral and dependant on the identity of their partner, hence they get engaged in both (insertive as well as receptive) roles during anal intercourse with another MSM^14^

^d^Kothi: Self-identified subgroup of MSM who are more effeminate and commonly play the receptive role during anal intercourse with another MSM^14^

Unadjusted bivariate analyses [unadjusted odds ratio (OR) and corresponding 95% confidence interval (95%CI)] revealed that participants from Kolkata had the highest odds of being HIV positive compared to MSM from other districts. Compared to the participants aged < 20 years, those with age 21–30 years and > 30years had respectively 1.44 times (OR: 2.44, 95% CI: 1.30–4.57) and 2.51 times (OR: 3.51, 95% CI: 1.61–7.64) higher chance of being HIV sero-positive. Laborers/servants (OR: 3.99, 95% CI: 1.16–13.70), small business-owners/cultivators (OR: 5.14, 95% CI: 1.47–17.98) and large business-owners/self-employed (OR: 5.33, 95% CI: 1.03–27.69) were 2.99, 4.14 and 4.33 times more likely to be HIV positive respectively, compared to their unemployed counterparts. With reference to MSM who visited the sentinel site for collecting condoms, those who came for official work (OR: 11.34, 95% CI: 2.65–48.44) and recreational (who visited the sentinel site drop-in-centers for the purpose of playing cards, carom or other games, watching television or getting engaged in other leisurely activities with other MSM) purpose (OR: 0.28, 95% CI: 0.09–0.86) respectively were 10.34 time more and 72% less likely to be HIV positive. Compared to the recruited subjects who didn’t exchange money for having sex with a man, paying (OR: 3.83, 95% CI: 1.66–8.81) and receiving money (OR: 2.24, 95% CI: 1.27–3.95) were associated with higher HIV risk (Table 2). Using the saturated model with all variables included, multivariate logistic regression [adjusted odds ratio (AOR)] revealed that compared to unemployed, all other occupations were likely to be at higher risk of HIV acquisition, although the analysis lacked power for students and service-holders. Controlling all other variables, transport workers (AOR: 8.95, 95% CI: 1.09–73.71), large business-owners/self-employed (AOR: 8.46, 95% CI: 1.25–57.49) and small business-owners/cultivators (AOR: 7.90, 95% CI: 1.67–37.38) had considerably higher odds of being HIV infected compared to unemployed participants. Alike the bivariate analysis, with reference to those who visited the sentinel sites for condom collection, visiting sentinel site for official work (AOR: 7.60, 95% CI: 1.21–47.83) and recreational purposes (AOR: 0.04, 95% CI: <0.01–0.36) were respectively more and less likely to be associated with HIV sero-positivity, after including all other variables in the model. Similar to the unadjusted association, after adjusting for all others, paying money for having sex with a man (AOR: 3.03, 95% CI: 1.10–8.33) was associated with higher HIV risk compared to those who didn’t exchange money for such purpose (Table 2).

**Table 2:** Unadjusted and adjusted^a^ association (using saturated model) of socio-demographic and behavioral characteristics with HIV sero-positivity among MSM attending HIV sentinel sites in West Bengal, 2010–11 (N = 1237).

Socio-demographic and behavioral characteristics	Categories	Frequency in each category (n)		Unadjusted			Adjusted			
			OR	95% CL^b^		p values	OR	95% CL^b^		p values
				Lower	Upper			Lower	Upper	
District (n = 1237)	Kolkata	250	Reference	Reference	Reference	Reference	Reference	Reference	Reference	Reference
	Hooghly	249	0.39	0.18	0.84	0.016*	0.68	0.21	2.24	0.523
	Burdwan	248	0.61	0.31	1.19	0.143	0.65	0.23	1.86	0.421
	Nadia	250	0.31	0.14	0.71	0.005*	5.63	0.69	45.83	0.106
	Darjeeling	240	0.24	0.10	0.60	0.002*	0.52	0.17	1.59	0.250
Age group (n = 1237)	Very young (15–20 yrs)	520	Reference	Reference	Reference	Reference	Reference	Reference	Reference	Reference
	Young (21–30 yrs)	570	2.44	1.30	4.57	0.006*	1.83	0.85	3.95	0.123
	Older (31–49 yrs)	147	3.51	1.61	7.64	0.002*	3.10	0.92	10.47	0.068
Current place of residence (n = 1235)	Urban	946	Reference	Reference	Reference	Reference	Reference	Reference	Reference	Reference
	Rural	289	1.02	0.57	1.86	0.937	1.29	0.58	2.86	0.527
Literacy status (n = 1236)	Illiterate	52	Reference	Reference	Reference	Reference	Reference	Reference	Reference	Reference
	Up to 5th standard	220	0.88	0.28	2.76	0.824	1.13	0.30	4.21	0.858
	6th to 10th standard	577	0.59	0.20	1.75	0.342	0.79	0.22	2.84	0.715
	11th to Graduation	362	0.56	0.18	1.73	0.310	0.70	0.17	2.86	0.614
	Post-graduation	25	0.50	0.05	4.72	0.545	0.52	0.05	6.02	0.600
Occupation (n = 1236)	Unemployed	163	Reference	Reference	Reference	Reference	Reference	Reference	Reference	Reference
	Laborer/servants	273	3.99	1.16	13.70	0.028*	5.13	1.10	23.94	0.038*
	Transport worker	29	3.95	0.63	24.75	0.142	8.95	1.09	73.71	0.042*
	Skilled/Semi-skilled worker	123	3.22	0.82	12.71	0.095	5.78	1.12	29.68	0.036*
	Student	289	1.13	0.28	4.58	0.863	3.17	0.59	17.14	0.180
	Small business/Cultivation	182	5.14	1.47	17.98	0.010*	7.90	1.67	37.38	0.009*
	Govt./Private service	144	2.73	0.69	10.74	0.152	3.02	0.54	16.93	0.208
	Large business/Self-employed	33	5.33	1.03	27.69	0.046*	8.46	1.25	57.49	0.029*
Duration of stay in current place of residence (in yrs) (continuous variable)			1.02	0.99	1.05	0.285	0.97	0.93	1.02	0.251
Reason for coming to the site (n = 1229)	Collect condoms	144	Reference	Reference	Reference	Reference	Reference	Reference	Reference	Reference
	For official work	10	11.34	2.65	48.44	0.001*	7.60	1.21	47.83	0.031*
	Drop in center/Meet other MSM	344	0.88	0.37	2.10	0.779	1.16	0.40	3.35	0.782
	Recreational purpose	314	0.28	0.09	0.86	0.026*	0.04	<0.01	0.36	0.004*
	Treatment/Counseling	417	1.27	0.57	2.85	0.561	1.21	0.48	3.08	0.691
Self-identified types (n = 1230)	Panthi	106	Reference	Reference	Reference	Reference	Reference	Reference	Reference	Reference
	Double decker	576	2.16	0.50	9.31	0.300	2.41	0.51	11.42	0.268
	Kothi	548	3.66	0.87	15.42	0.078	4.09	0.83	20.19	0.084
Days since last sex with a man			1.01	0.99	1.03	0.304	1.00	0.98	1.03	0.900
Had sex with female in last 6 mths (n = 1235)	No	385	Reference	Reference	Reference	Reference	Reference	Reference	Reference	Reference
	Yes	850	1.48	0.82	2.67	0.198	1.15	0.53	2.47	0.725
Received/paid money for having sex with a man in last 1 mth (n = 1237)	No	750	Reference	Reference	Reference	Reference	Reference	Reference	Reference	Reference
	Paid money	64	3.83	1.66	8.81	0.002*	3.03	1.10	8.33	0.032*
	Received money	311	2.24	1.27	3.95	0.005*	1.32	0.59	2.93	0.502
	Both	112	0.99	0.34	2.89	0.988	1.01	0.32	3.23	0.989

^a^ Each socio-demographic and behavioral characteristic adjusted for all others.

^b^ CL: confidence limits

* p value indicates statistically significant association with HIV sero-positivity at α = 0.05

In the final model for multivariate logistic regression we controlled for age, current place of residence, literacy status, occupation, years of stay at current place of residence and reason for coming to the sentinel site. Using this model, Kothis (with reference to Panthis, AOR: 4.64, 95% CI: 1.03–20.89), those who paid (AOR: 2.96, 95% CI: 1.15–7.58) or received (AOR: 2.06, 95% CI: 1.06–3.99) money (compared to those who didn’t exchange money) for having sex with a man were found to have much higher odds of being HIV positive (Table 3).

**Table 3:** Adjusted^a^ association (using parsimonious model) between sexual behavior and HIV sero-positivity among MSM attending HIV sentinel sites in West Bengal, 2010–11 (N = 1237).

Sexual behavior	Categories	OR	95% CL^b^		p values
			Lower	Upper	
Self-identified types	Panthi	Reference	Reference	Reference	Reference
	Double decker	2.40	0.53	10.84	0.255
	Kothi	4.64	1.03	20.89	0.046*
Had sex with female in last 6 mths	No	Reference	Reference	Reference	Reference
	Yes	1.67	0.88	3.19	0.118
Received/paid money for having sex with a man in last 1 mth	No	Reference	Reference	Reference	Reference
	Paid money	2.96	1.15	7.58	0.024*
	Received money	2.06	1.06	3.99	0.033*
	Both	0.96	0.32	2.92	0.944
Days since last sex with a man (as continuous variable)		1.00	0.98	1.03	0.698

^a^ Adjusted for age group, current place of residence, literacy status, occupation, duration of stay in current place of residence and reason for coming to the service point

^b^ CL: confidence limits

* p value indicates statistically significant association with HIV sero-positivity at α = 0.05

## Discussion

HIV positivity among MSM varied considerably (0.36% to 14.98%) across states in India [7,11,14,17,18]. Social stigma and ill-defined sexual identity might be responsible for existing discrepancies in the estimates of HIV burden associated with this largely hidden community. The utmost concern is the possibility of underestimation of the true burden of HIV in this hard-to-reach population. It is evident from prior research that MSM had consistent elevated risk for HIV compared to general population [19]. This study involving MSM in West Bengal found HIV sero-positivity of 5.09% which is nearly 18 times the estimate in the general population.

Mean age of 23.4 years of the participants corroborates with the findings of other studies that comparatively younger MSM are taking part in HSS [7,9,11,18]. Increasing age was associated with higher HIV risk which supported similar observations by Setia et al and Hernandez et al [18,20] among MSM attending STI clinics in Mumbai. Older MSM, who are at higher risk of HIV, need appropriate intervention. Majority (76.6%) participants were urban residents. In a survey among MSM in Andhra Pradesh during 2003–04, only 14.5% of the participants were from rural areas [9]. HIV prevalence was found to be higher among MSM in urban areas compared to their rural counterparts during 2008–09 [21]. Majority participation from urban areas may be explained by possibilities that in most occasions the HSS sites are found to be located in urban areas. Keeping both the HIV and MSM related taboo and stigma in mind, it is quite expected that due to social issues and geographical locations, participation of rural MSM will be less. In the era of changing dynamics of HIV epidemic in India, although being predominantly urban, the epidemic now gradually invading rural areas of the country. Hence, to understand the burden and correlates of HIV in those areas, studies on rural MSM should be emphasized.

The mean duration of stay at the current place was 22.2 years. This may indicate that MSM who were permanent residents of their current place of residence for longer period were availing services more probably because they were being reached easily as their network became wider with time. MSM, who are either migrants or new to an area are required to be studied also to understand their HIV risk and associated factors. Only 4.21% of the study participants were illiterate and majority had at least high school level education. This contradicts some of the prior observations among MSM in India, where majority were found to be illiterate or have education up to primary level [22]. Although there was no association between literacy status and HIV risk in our analyses, but MSM attending voluntary counseling and testing services in Mumbai during 2003–04 with no formal education were at higher risk of HIV [17]. The main concern is that poor literacy among MSM might be making them less aware about HIV and their self-perceived risk of acquiring HIV infection might be low which could result in their less participation [18,23].

High HIV risk was observed among large business owner/self-employed (highest risk) and transport workers (4^th^ highest risk) but their proportions among the participants were only 2.67% and 2.35% respectively. Proportions of the skilled/semiskilled workers were also low (9.95%) which contradicted the observation by Setia et al [18]. Being in service was not found to be associated with higher risk, which again is not in agreement with the national estimate of the 2008–09 country report [21]. We found that unemployed MSM were at lower HIV risk which corroborated with report by Gupta et al [22]. Distribution and HIV risk across the strata of occupation might be different among MSM in West Bengal compared to other Indian states.

The highest HIV risk was observed among those who visited the sentinel site for official work. This group was only 0.81% of the total participants. Being involved in official work at the site might have helped them to be economically more stable and having wider network which in turn might have increased their sexual risk behavior. Being identified as an MSM is associated with immense social stigma and discrimination in India. As a result this population remains largely hidden. Among self-identified MSM subtypes, Panthis and Double-deckers are more hidden than Kothis. Being identified as an MSM often results in huge stigma in Indian society. Feminine appearance and behavior of Kothis make them easily identifiable and thus easily discriminated as opposed to masculine Panthis and Double-deckers who remains more hidden [24, 25]. This explains why in most of the studies in India majority of the participants were Kothis [20,22,26]. They were also more likely to show up in investigations regarding willingness to get tested for HIV [27] or participation in vaccine trials [28] despite facing stronger barriers for accessing preventive and curative services [29, 30]. In our study also the proportion of Panthis among participating MSM was as less as 8.62% only. But Double-deckers were as high as 46.83% signifying the ability of HSS in West Bengal in recruiting diverse sample of MSM population in terms of self-identified subgroups. Burden of other risk behaviors like alcoholism is found to be relatively less among Kothis [31]. Still, for being mostly receptive, the HIV acquisition risk is likely to be higher among Kothis compared to Panthis [11,32] as also observed in this study. The existing discrepancies in HIV prevalence observed among subsets of MSM may be due to non-existence of uniform sexual identity and role leading to considerable overlapping of sexual orientation among self-identified MSM. Heterosexual activities were reported by 68.8% of MSM. This typically corroborates with prior studies [9,11,18]. Although according to our results, such activity seemed to be associated with higher HIV risk, but we didn’t have sufficient power of the analysis. Majority of the MSM in India probably do not have an overriding distinct homosexual identity and under social pressure they mix well with female partners for sex. Thus, they are probably acting as a bridge population spreading HIV from other high risk MSM group to their regular female partner or spouses [9,11,14,15,17,33,34]. Mean duration since last sex with a man was calculated to be 8.4 days in our analysis and we didn’t find any association between this and HIV positivity. In our study 60.6% MSM reported that they didn’t exchange money for having sex with a man in last one month. Having received or paid money for having sex with a man was found to be strongly associated with HIV sero-positivity. Although previous studies reported this behavior to be much more common among MSM, association with HIV risk was found to be similar [11,14,22,35]. Prior studies on MSM revealed a positive association between being IDU and HIV sero-positivity [14]. In our study nobody reported IDU. Further research might unfold the possible explanations including under-reporting or non-existence of such practices among MSM in this part of India.

Study participants were those who visited HSS sites for availing intended services offered, so they may be more active, sick and visible MSM and thus might not be a representative sample of MSM in West Bengal, therefore, generalizability of the results might be limited to its perspective. There was also a possibility of selection bias if participation got affected by their HIV status and socio-behavioral characteristics, but the chances of selection bias were comparatively less as consecutive sampling strategy was followed wherein even known HIV positive status was not an exclusion criterion. Behavioral information (history of sex with female, days since last sex with man, paid or received money for sex with a man, use of injectable drugs) were all self-reported and degree to which social desirability and accuracy of memory influenced these responses might vary. The social desirability bias due to under reporting of high risk behaviors is expected to be minimized because of anonymity and confidentiality of the provided information. Being cross-sectional by design, the direction and magnitude of associations so obtained in this study may not be indicative of causality. Moreover, exclusion of other potential factors t might have influenced risky sexual behaviors.

## Conclusions

MSM of older age, being a transport worker or large business owner/self-employed, visiting sentinel site for official work, being a Kothi, having received/paid money for sex with a man were all found to be strongly associated with higher HIV risk among MSM who participated in 2010–11 round of HSS in West Bengal. In spite of limitations of generalizability, social desirability and other potential factors this study, for the first time, presenting a baseline document providing useful information on the role of socio-behavioral correlates of HIV acquisition among MSM in West Bengal which might help in formulating policies and intervention programs concerning MSM, who appeared to be both understudied and underserved in Indian society.
